# SOX2 recruits KLF4 to regulate nasopharyngeal carcinoma proliferation via PI3K/AKT signaling

**DOI:** 10.1038/s41389-018-0074-2

**Published:** 2018-08-15

**Authors:** Jianming Tang, Guansheng Zhong, Jianhui Wu, Haiyan Chen, Yongshi Jia

**Affiliations:** 1 Department of Radiation Oncology, Zhejiang Provincial People’s Hospital, People’s Hospital of Hangzhou Medical College, Hangzhou, Zhejiang 310014 P.R. China; 2 Department of Thyroid and Breast Surgery, Zhejiang Provincial People’s Hospital, People’s Hospital of Hangzhou Medical College, Hangzhou, Zhejiang 310014 P.R. China; 3 Department of The Otolaryngology, Meizhou People’s Hospital, Meizhou, Guangdong 514000 P.R. China; 4 Department of Radiation Oncology, The Second Affiliated Hospital of Zhejiang University School of Medicine, Hangzhou, Zhejiang 310009 P.R. China

## Abstract

SOX2 is a transcription factor that contributes to transcription modification and cancer, but the mechanism by which SOX2 regulates nasopharyngeal carcinoma cell proliferation is not well understood. Here, we identify a SOX2 signaling pathway that facilitates nasopharyngeal carcinoma, where it is upregulated. SOX2 expression was associated with nasopharyngeal carcinoma patient survival. SOX2 knockdown inhibited cell proliferation, colony formation, and tumorigenesis in an subcutaneous mouse xenograft model system. Six hundred and ninety-nine candidate SOX2 downstream dysregulated genes were identified in nasopharyngeal carcinoma cells through cDNA microarray analysis. SOX2 recruited the nuclear transcription factor KLF4 to bind to the PIK3CA promoter upregulate PIK3CA expression, acting to enhance PI3K/AKT signaling and tumorigenesis by upregulating PIK3CA expression. Besides, overexpressing activated AKT or PIK3CA rescued the growth inhibition of cells due to SOX2 knockdown. Together, our study suggest that SOX2 exhibits oncogenic properties and may be a reliable molecular biomarker in nasopharyngeal carcinoma. Targeting SOX2 might be a promising treatment strategy for nasopharyngeal carcinoma treatment.

## Introduction

Nasopharyngeal carcinoma, a most commonly diagnosed cancer originating from the epithelial lining of the nasopharynx, has remained high incidence rate in endemic regions, especially in southern China, northern Africa, and Alaska^1^. Accumulating evidence from human and animal studies suggests that genetic factors, environmental factors, and the Epstein-Barr virus (EBV) infection have played critical roles in nasopharyngeal carcinoma progression^2–5^. Although recent anti-EBV antibodies and plasma EBV DNA contribute to the advancement of diagnosis and prognosis prediction in nasopharyngeal carcinoma, the clinical treatment outcome is still unsatisfactory^6^.

On account of non-ideal therapeutic approach and the unsatisfactory outcome of nasopharyngeal carcinoma patients, there is an urgent need to identify reliable early diagnostic biomarkers as well as novel therapeutic targets for those patients. A growing body of evidence has implicated that transcription factors are involved in tumorigenesis through regulating the abnormal expression of tumor-related genes. However, the precise molecular mechanisms that account for it remain unclear.

Sex determining region Y-box 2 gene (SOX2), located at chromosome 3q26.33, encodes a transcription factor which contains a high mobility group DNA-binding domain in its structure^7^. Functionally, SOX2 maintains stem cell pluripotency and determines cell fate. However, it is becoming increasingly apparent that ectopic expression and amplification of SOX2 was significantly correlated with cancer development^7^. Increasing evidence strongly suggests that SOX2 is implicated in regulating the development of various malignant tumors, including glioblastoma, small-cell lung cancer, and nasopharyngeal carcinoma^8–10^. SOX2 was found to bind long non-coding RNA *ANRIL* in nasopharyngeal carcinoma^10^. It has been reported that SOX2 works as transcriptional activators in reprogramming human fibroblasts^11^. SOX2 is also shown as a regulator of PI3K/AKT signaling in spermatogonial stem cells^12^. Moreover, SOX2 was reported to promote tumorigenesis, progression, and metastasis in multiple types of cancer through controlling stem cell activity^13,14^. However, further explorations are required to elucidate the functions of SOX2 in tumor progression, including nasopharyngeal carcinoma.

Krüppel-like factor 4 (KLF4), also known as zinc-finger transcription factor, is reported to be expressed mainly in postmitotic, terminally differentiated epithelial cells in organs such as skin and gastrointestinal tract, playing a vital role in the development and cell differentiation^15–17^. Recently, emerging evidences have indicated that KLF4 functions as a tumor suppressor in various tumors^18–20^. Conversely, conflicting results also reveal that KLF4 is overexpressed in primary breast and other cancers, functioning a oncogenic role in tumor development and progression^21,22^. However, the clear mechanism by which KLF4 exerts in nasopharyngeal carcinoma remains unknown.

In the current study, we found that SOX2 was genetically overexpressed both in clinical nasopharyngeal carcinoma tissues and cell lines. Both in vitro and in vivo studies showed that SOX2 exhibits a critical oncogene to regulate proliferation and tumorigenesis in nasopharyngeal carcinoma. Furthermore, we successfully demonstrated that KLF4 serves as a oncogene and directly binds to SOX2 in nasopharyngeal carcinoma. Finally, we examined the mechanisms by which SOX2 regulates nasopharyngeal carcinoma tumorigenesis. Importantly, our data demonstrate that the interaction of KLF4 and SOX2-induced expression of PI3K/AKT signaling is a critical mechanism for SOX2-driven nasopharyngeal carcinoma proliferation.

## Results

### SOX2 expression is prognostic for clinical nasopharyngeal carcinoma

**Fig. 1:**
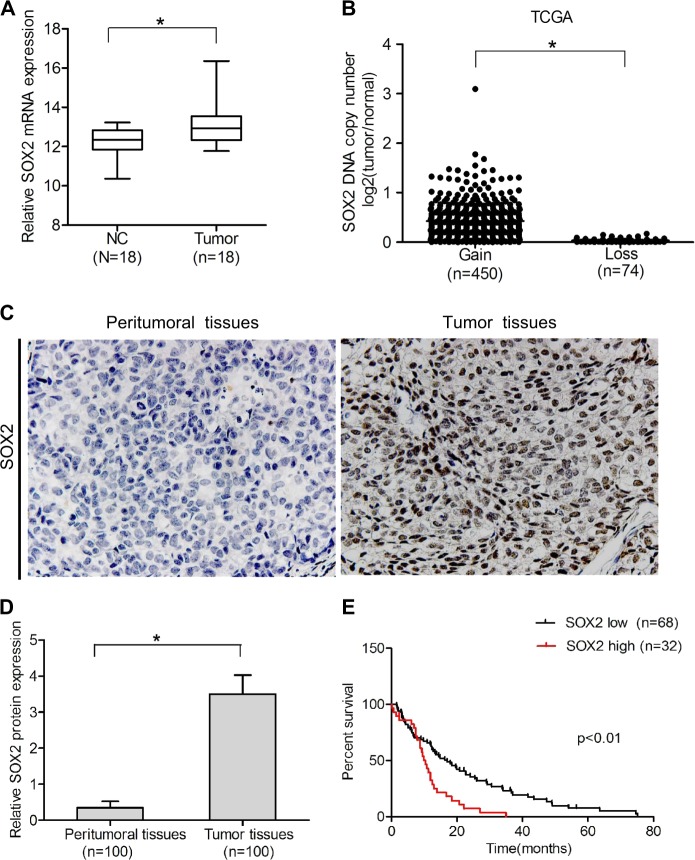
SOX2 expression is prognostic for clinical nasopharyngeal carcinoma. **a** Expression levels of SOX2 mRNA are significantly higher in nasopharyngeal carcinoma samples compared with normal nasopharyngeal tissues. Expression data of SOX2 mRNA were downloaded from the GSE53819 dataset. **b** Expression levels of SOX2 DNA copy number are significantly higher in head and neck samples compared with normal tissues from a TCGA data sets. **c** IHC staining of SOX2 in clinical nasopharyngeal tissues and peritumoral tisssues specimens. **d** Quantitative analysis of SOX2 protein expression in **c**. **e** Kaplan–Meier analysis of patients with high SOX2 protein-expressing nasopharyngeal carcinoma versus low SOX2 protein-expressing nasopharyngeal carcinoma. Statistical analysis was performed by log-rank test in a GraphPad Prism version 5.0 for Windows. Error bars ± SD. *P < 0.05. Data are representative from two independent experiments

In order to determine the role of SOX2 in nasopharyngeal carcinoma, the expression level of SOX2 in clinical specimens of patients was examined. We downloaded one nasopharyngeal carcinoma microarray data sets, GSE53819, and examined SOX2 mRNA expression in clinical nasopharyngeal carcinoma samples and normal nasopharyngeal tissues. As shown in Fig. 1a, SOX2 was markedly overexpressed in nasopharyngeal carcinoma compared with non-cancerous nasopharyngeal tissue. We also analyzed the copy numbers of SOX2 in TCGA head and neck dataset from http://xena.uscs.edu/public-hubs, and found that compared with normal nasopharyngeal carcinoma tissues, 86% (450/524) tumors were amplified (log2 > 0, tumor versus normal) (Fig. 1b).

Then, we performed immunohistochemistry (IHC) staining assays in the total 100 pairs of clinical nasopharyngeal carcinoma tissues and their matched peritumoral tissues. As shown in Fig. 1c, d, SOX2 staining was negative or weak in peritumoral tissues. In contrast, SOX2 was expressed strongly in the nasopharyngeal carcinoma tissues, suggesting that the levels of SOX2 expression are upregulated in tumor tissues.

Finally, we examined the relationship of SOX2 expression and nasopharyngeal carcinoma patient survival by Kaplan–Meier survival analysis. As shown in Fig. 1e, Kaplan–Meier survival analysis revealed a statistically significant worse prognosis for nasopharyngeal carcinoma patients with high SOX2 protein levels compared with those with low. Together, these results strongly indicate that upregulation of SOX2 was closely associated with tumor progression and poor prognosis in nasopharyngeal carcinoma patients.

### Knockdown of SOX2 inhibits nasopharyngeal carcinoma cell proliferation, colony formation, and tumor growth

**Fig. 2:**
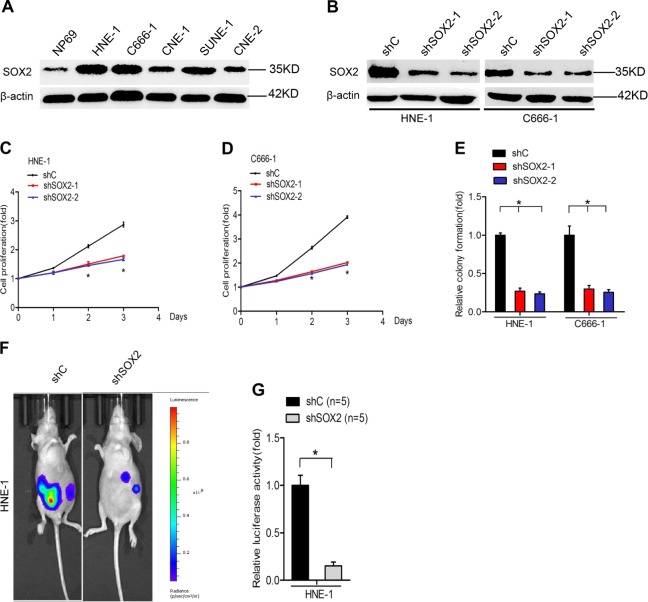
Knockdown of SOX2 inhibits nasopharyngeal carcinoma cell proliferation, colony formation, and tumor growth. **a** Western blotting analysis of SOX2 protein expression in nasopharyngeal carcinoma cells and normal nasopharyngeal cells. Actin was used as a control. **b** Western blot analysis of SOX2 knockdown in HNE-1 and C666-1 cells. **c**, **d** Effects of SOX2 knockdown on nasopharyngeal carcinoma cell proliferation. **e** Effects of SOX2 knockdown on nasopharyngeal carcinoma cell colony formation. **f** Representative bioluminescence images of SOX2 knockdown-inhibited HNE-1 subcutaneous tumor generation. Mice were imaged at 3–4 weeks after implantation. Data were from two independent experiments with five mice per group with similar results. **f** Quantification of the bioluminescence activity in **e**. Error bars ± SD. *P *<* 0.05. Data are representative from two independent experiments

To demonstrate the role of SOX2 in nasopharyngeal carcinoma tumorigenesis, we first compared the expression of SOX2 in C666-1, SUNE-1, HNE-1, CNE1, and CNE2 nasopharyngeal carcinoma cell lines and NP69 normal nasopharyngeal cell line. We found SOX2 expression was markedly overexpressed in nasopharyngeal carcinoma cell lines compared with NP69 cells (Fig. 2a), suggesting that higher SOX2 expression may be associated with nasopharyngeal carcinoma phenotype. Subsequently, lentivirus-mediated short hairpin RNAs (shRNA) of SOX2 or a control shRNA was used to deplete SOX2 in HNE-1 and C666-1 nasopharyngeal carcinoma cell lines (Fig. 2b). As shown in Fig. 2c–e, knockdown of endogenous SOX2 significantly suppress cell proliferation and colony formation in both nasopharyngeal carcinoma cell lines.

In order to further determine whether SOX2 is critical for nasopharyngeal carcinoma tumorigenesis, we employed an subcutaneous nasopharyngeal carcinoma model. HNE-1 nasopharyngeal carcinoma cells transfected with shSOX2, or shControl were implanted subcutaneously to generate nasopharyngeal carcinoma xenografts in immunocompromised mice. Then, the effects of SOX2 depletion on nasopharyngeal carcinoma tumorigenesis were assessed. Compared with the control xeno-graftmodels, knockdown of SOX2 significantly inhibited nasopharyngeal carcinoma tumor growth (Fig. 2f, g). Above, data definitely support that SOX2 is critical for cell proliferation, and tumor growth in nasopharyngeal carcinoma.

### Identification of SOX2 downstream candidate genes in nasopharyngeal carcinoma

**Fig. 3:**
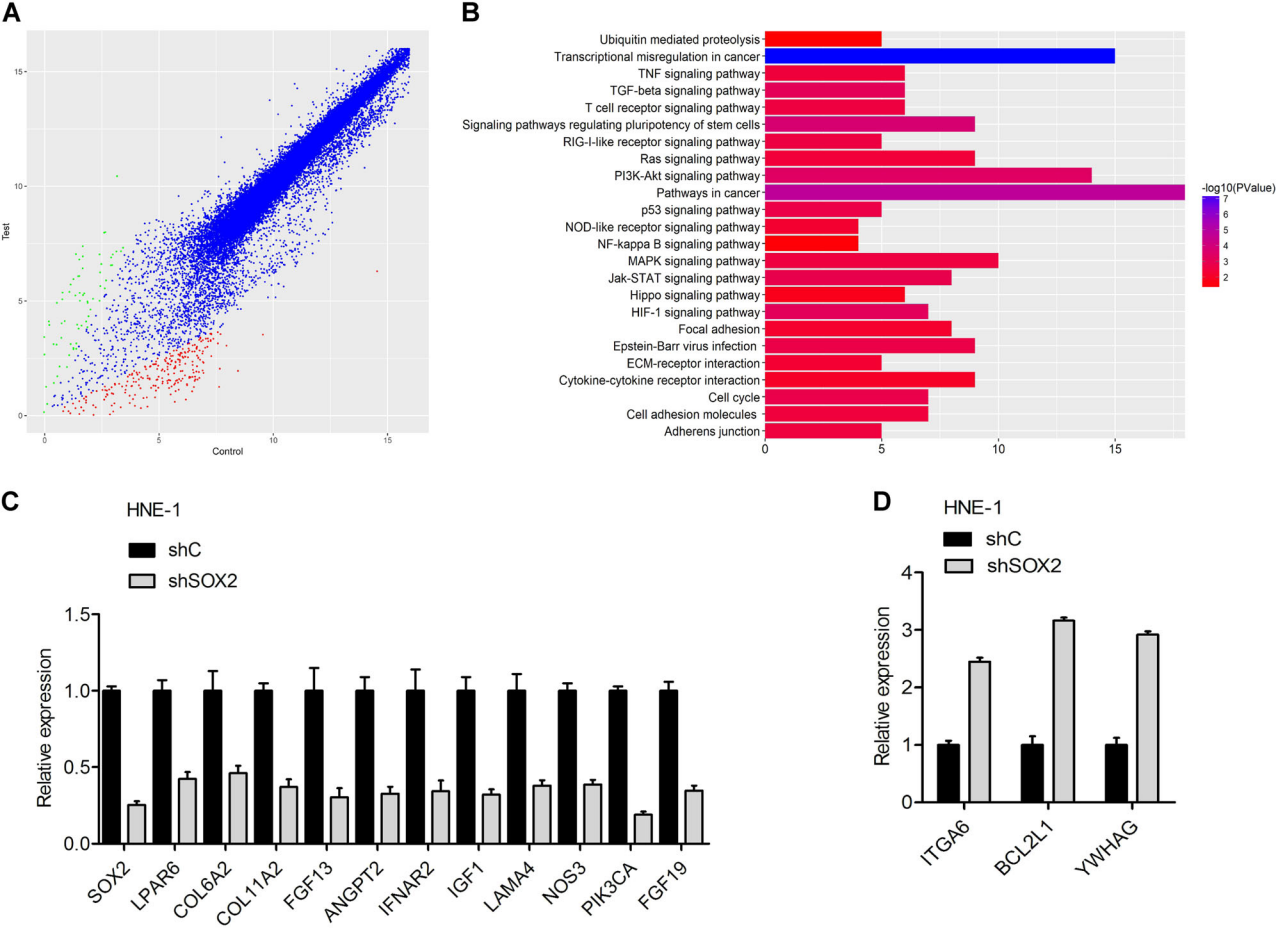
Identification of SOX2 downstream candidate genes in nasopharyngeal carcinoma. **a** Scatter plot of 23230 gene expression levels (log2) in HNE-1 cells transfected with SOX2 shRNA (*y-*axis) compared with the control group (*x-*axis). Green dots, set of genes exhibiting significant upregulation upon SOX2 knockdown; red dots, set of genes exhibiting significant down-regulation upon SOX2 knockdown; black dots, the genes without significant change. **b** The signaling pathways response upon SOX2 knockdown by Gene KEGG analyses. **c**, **d** mRNA expression of eleven down-regulation genes (**c**) and three upregulation genes (**d**) was confirmed by real-time PCR analysis. Error bars ± SD. Data are representative from two independent experiments

To further study underlying molecular mechanisms, we performed a microarray gene expression analysis in HNE-1 cell line transfected with shSOX2. HNE-1 cell line transfected with shControl were used as the control. Among all the 23230 analyzed unique genes, a total of 699 genes were found to be differentially regulated, of which 212 were upregulated and 487 downregulated (Fig. 3a).

To fully investigate the signaling pathways responding to SOX2 knockdown, we performed Gene KEGG pathway analyses. The significant genes implicated pathways regulated by SOX2 had relation with cancer-related pathways including PI3K–AKT (Fig. 3b and Supplementary Table 2). Next, we performed a further identification of the significant genes involved in PI3K–AKT pathway based on the KEGG analyses (Supplementary Table 3). As shown in Fig. 3c, d, the mRNA expressions of 11 genes (LPAR6, COL6A2, COL11A2, FGF13, ANGPT2, IFNAR2, IGF1, LAMA4, NOS3, PIK3CA, and FGF19) among 14 genes were detected to be downregulated by quantitative reverse transcriptase polymerase chain reaction (qRT-PCR) analysis, while ITGA6, BCL2L1, and YWHAG were found upregulated. Therefore, the above data strongly suggest that SOX2 regulates PI3K–AKT signaling pathway.

### SOX2 regulates AKT activity

**Fig. 4:**
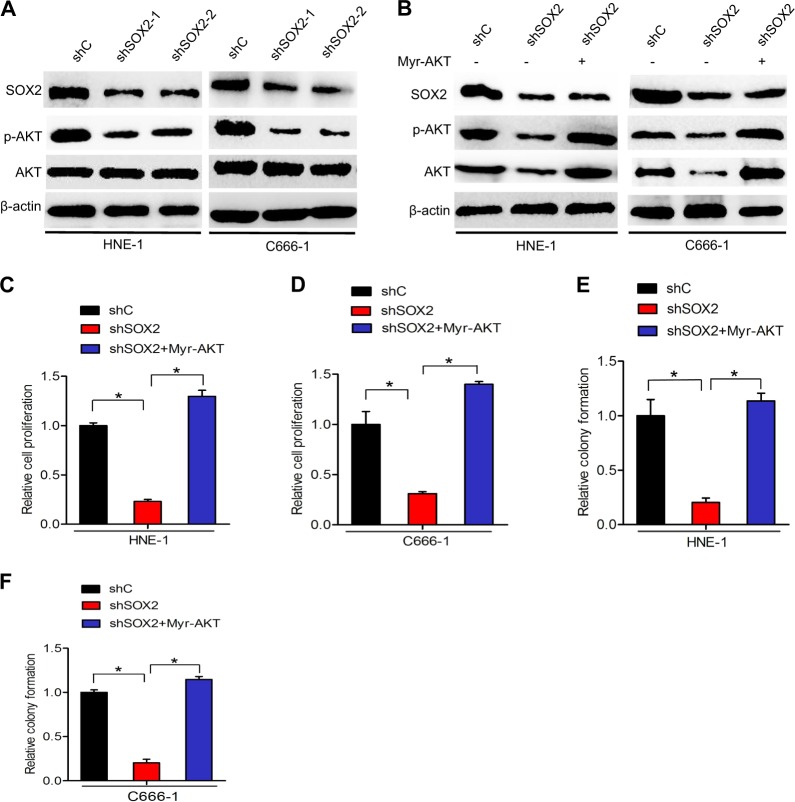
SOX2 regulates AKT activity. **a** Western blot analysis of effects of SOX2 knockdown on AKT activation. **b** Effects of overexpression of a constitutively activated Akt (Myr-AKT) mutanton on SOX2 knockdown-inhibited Akt activation. **c**, **d** Effects of overexpression of Myr-AKT on SOX2 knockdown-inhibited nasopharyngeal carcinoma cell proliferation. **e**, **f** Effects of overexpression of Myr-AKT on SOX2 knockdown-inhibited nasopharyngeal carcinoma cell colony formation. Error bars ± SD. *P < 0.05. Data are representative from two independent experiments

As AKT activity plays a crucial role in the function of PI3K–AKT signaling^23^, we determined the effect of SOX2 knockdown on phosphorylation of AKT (p-AKT) in HNE-1 and C666-1 nasopharyngeal carcinoma cells. As shown in Fig. 4a, knockdown of SOX2 markedly decreased AKT phosphorylation, suggesting that SOX2 regulates AKT activity in nasopharyngeal carcinoma.

To support that SOX2 mediates AKT activity, we overexpressed a constitutively activated (CA) AKT (Myr-AKT) mutant in HNE-1-shSOX2 and C666-1-shSOX2 cell lines in which SOX2 was stably knocked down. Compared with the control, SOX2 knockdown in HNE-1 and C666-1 cells inhibited AKT phosphorylation (Fig. 4b), cell proliferation (Fig. 4c, d), and colony formation (Fig. 4e, f). Importantly, overexpression of the CA AKT mutant in HNE-1 and C666-1/shSOX2 cells markedly restored AKT phosphorylation (Fig. 4b), cell proliferation(Fig. 4c, d), and colony formation (Fig. 4e, f), which were inhibited by SOX2 knockdown. Overall, these data demonstrate that SOX2 regulates AKT activity to promote nasopharyngeal carcinoma cell proliferation.

### SOX2 upregulates PIK3CA expression in nasopharyngeal carcinoma to activate PI3K/AKT signaling

**Fig. 5:**
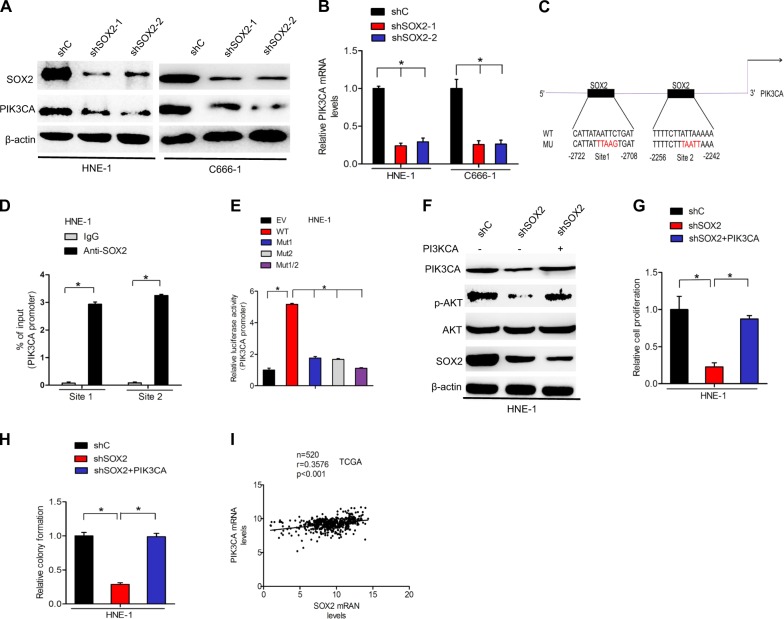
SOX2 upregulates PIK3CA expression in nasopharyngeal carcinoma to activate PI3K/AKT signaling. **a** Effects of SOX2 knockdown on PIK3CA protein expression in HNE-1 and C666-1 cells. **b** qRT-PCR analysis of effects of SOX2 knockdown on PIK3CA mRNA expression. **c** Schematic diagram of putative SOX2-binding sites in PIK3CA promoter. **d** ChIP assays on two SOX2-binding sites of PIK3CA promoter were performed in CNE2 and HNE-1 cells. **e** Analysis of the relative luciferase activity in HNE-1 cells cotransfected with wild type or the various mutant PIK3CA promoters. **f** Overexpression of PIK3CA rescues SOX2 knockdown-inhibited AKT activation. **g** Overexpression of PIK3CA rescues SOX2 knockdown-inhibited cell proliferation. **h** Overexpression of PIK3CA rescues SOX2 knockdown-inhibited cell colony formation. **i** Correlation of expression of PIK3CA with SOX2 in head and neck patients from a TCGA data sets. Error bars ± SD. *P < 0.05. Data are representative from two independent experiments

It is well known that PIK3CAan, oncogene encoding the p110a catalytic subunit of the phosphoinositide 3-kinase (PI3K), is frequently overexpressed in malignant tumors, which induce the aberrant activation of the PI3K/AKT signaling pathway^24^. To further investigate the mechanisms underlying the effects of SOX2 knockdown, the expression of PIK3CA in HNE-1 and C666-1 cell lines was assessed by qRT-PCR and western blotting. As shown in Fig. 5a, b, SOX2 knockdown markedly inhibited PIK3CA protein and mRNA expression levels in both HNE-1 and C666-1 cells. Importantly, we performed in silico analysis to predict the putative transcription factors binding to the promoter of PIK3CA (http://jaspar.genereg.net/). Two putative SOX2-binding sites were found in the promoter of PIK3CA at −2722 to −2708 and −2256 to −2242 sites (Fig. 5c). To reveal whether SOX2 binds to the promoter of PIK3CA at these sites, we performed ChIP-qPCR assays in HNE-1 and C666-1 using primers that flank a 220-bp region containing SOX2-binding site 1, or a 230-bp region containing SOX2-binding site 2 in the PIK3CA promoter. As shown in Fig. 5d, endogenous SOX2 protein bound with both site 1 region and site 2 region. To further validate this finding that SOX2 binds to the promoter of PIK3CA, we performed promoter luciferase assays. PIK3CA wild-type promoter significantly promoted luciferase activity compared with the empty vector, whereas mutation of site 1, site 2, both of them significantly decreased PIK3CA promoter luciferase activity (Fig. 5e). These data suggested that SOX2 mediated PIK3CA expression to regulate nasopharyngeal carcinoma cell proliferation.

To further validate that SOX2 upregulates PIK3CA expression thereby activating PI3K/Akt signaling pathways in nasopharyngeal carcinoma, we overexpressed PIK3CA in HNE-1 nasopharyngeal carcinoma cells with SOX2 shRNAs or a control shRNA. Overexpression of the PIK3CA in HNE-1-shSOX2 cells markedly restored AKT phosphorylation (Fig. 5f), cell proliferation(Fig. 5g), and colony formation (Fig. 5h) inhibited by SOX2 knockdown. Moreover, we evaluated the expression of SOX2 and PIK3CA in the 520 head and neck tumors with RNA sequencing data available from TCGA. Impressively, SOX2 expression was significantly correlated with the expression of PIK3CA in these samples (Fig. 5i).

### KLF4 interacts with SOX2 and acts as a oncogene in nasopharyngeal carcinoma

**Fig. 6:**
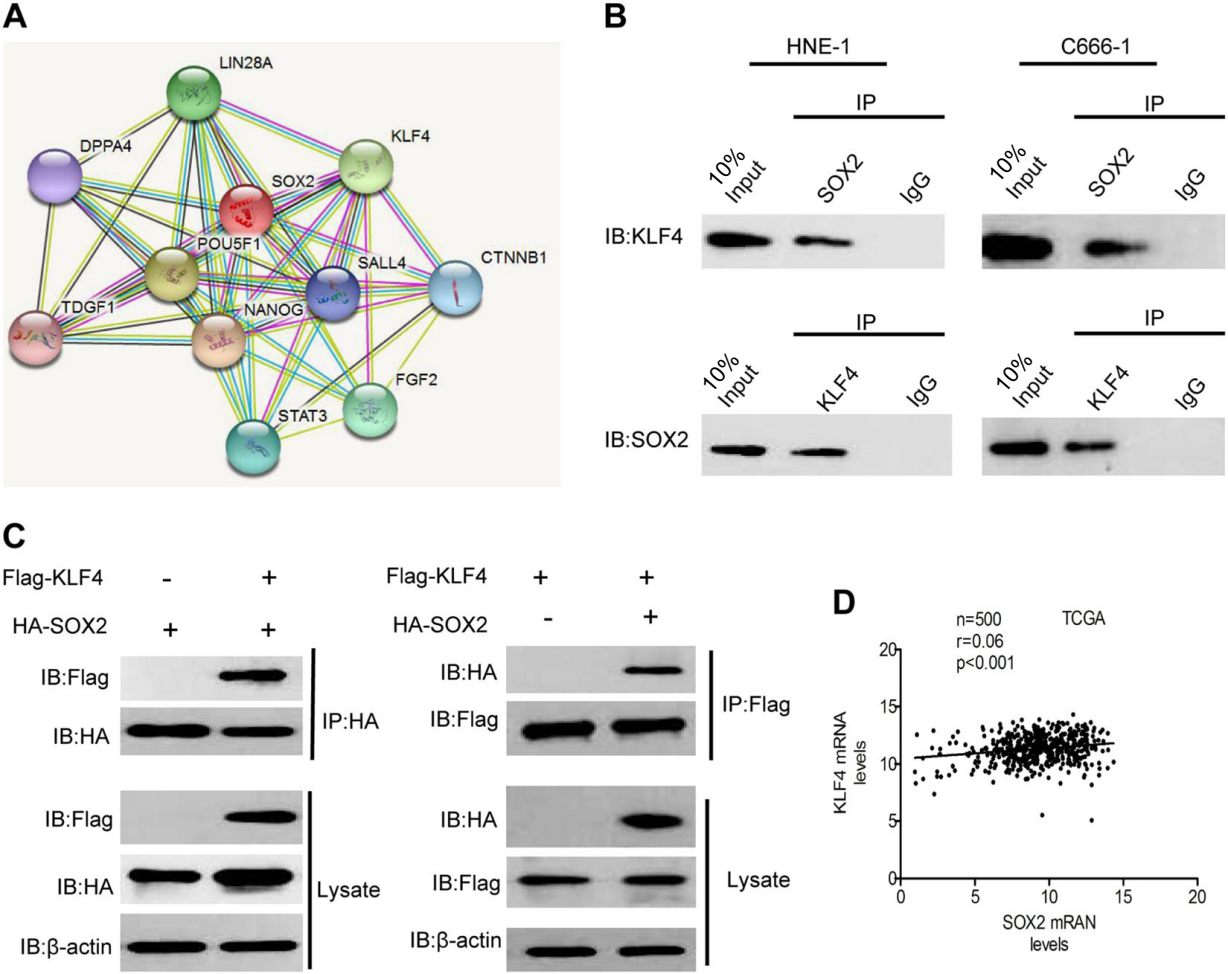
SOX2 interacts with KLF4. **a** Analysis of the direct interaction of SOX2 and KLF4 by String Prediction. **b** Cellular extracts of CNE2 and HNE-1 cells were subjected to IP using SOX2 antibody or control IgG, followed by IB with KLF4 antibody (upper panels). Reciprocal IP was done using KLF4 antibody or control IgG, followed by IB with the SOX2 antibody (lower panels). **c** 293T cells were cotransfected with Flag-KLF4 and HA-SOX2 plasmids. Cellular extracts were subjected to IP and IB analyses with the indicated antibodies. **d** Correlation of expression of KLF4 with SOX2 in head and neck patients from a TCGA data sets. Error bars ± SD. Data are representative from two independent experiments

Since SOX2 had been reported to work with KLF4 as transcriptional activators in reprogramming human fibroblasts^11^, we assessed whether KLF4 directly interacts with SOX2 in nasopharyngeal carcinoma. As shown in Fig. 6a, we performed an analysis of the direct interaction of SOX2 and KLF4 (https://string-db.org/cgi/input.pl).

Endogenous KLF4 bound to endogenous SOX2 was determined by co-IP assays (Fig. 6b). The interaction between KLF4 and SOX2 was further confirmed by immunoprecipitation (IP) analysis of 293T cells transfected with HA-tagged SOX2 and Flag-KLF4 (Fig. 6c). Since KLF4 can act as both a tumor enhancer and suppressor, we compared expression of KLF4 in tumor tissues with non-cancerous tissues to determine its exactly role in nasopharyngeal carcinoma. We found that KLF4 markedly upregulated both in samples from TCGA and fresh clinical tumor tissues (Supplementary Fig. 1A and B). Similar overexpression of KLF4 was also observed in various nasopharyngeal carcinoma cell lines (Supplementary Fig. 1C). By consistently knocking down expression of KLF4 in HNE-1 and C666-1 cell lines, we demonstrated that depletion of KLF4 significantly inhibited nasopharyngeal carcinoma growth both in vitro and in vivo (Supplementary Fig. 2). To further assess the clinic correlation of KLF4 and SOX2, we evaluated the expression of KLF4 and SOX2 in the 500 head and neck tumors with RNA sequencing data available from TCGA. As shown in Fig. 6d, the SOX2 expression was significantly correlated with the expression of KLF4 in these samples. Taken together, these results clearly indicated that KLF4 could directly binds to SOX2 and might serve as a oncogenic molecular prognostic marker for nasopharyngeal carcinoma.

### SOX2 increases PIK3CA expression by enhancing KLF4 binding to the PIK3CA gene promoter

**Fig. 7:**
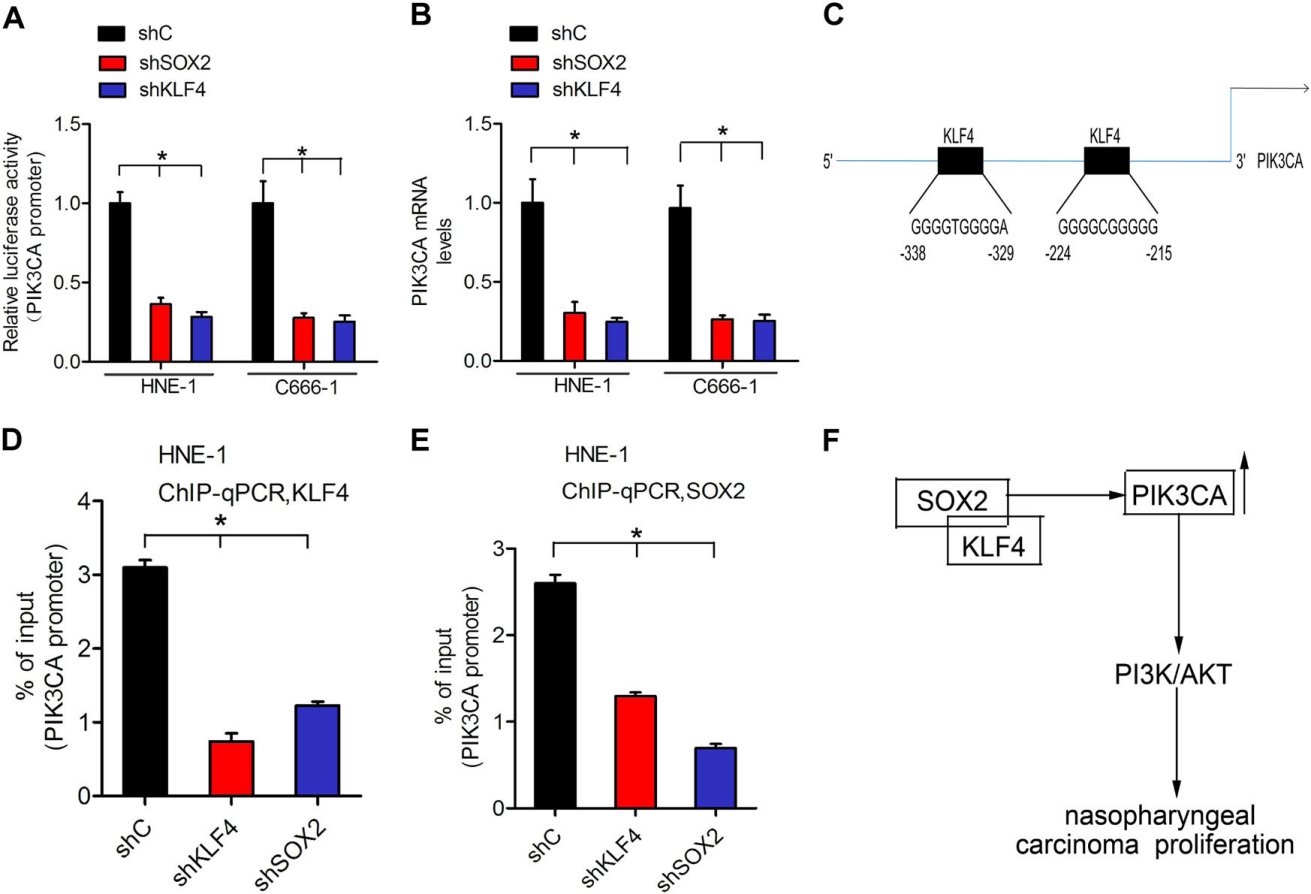
SOX2 increases PIK3CA expression by enhancing KLF4 binding to the PIK3CA gene promoter. **a** PIK3CA promoter luciferase reporter was transfected into HNE-1 and C666-1 cells expressing shcontrol, shKLF4, and shSOX2. **b** qRT-PCR analysis of PIK3CA mRNA expression in HNE-1 and C666-1 cells expressing shcontrol, shKLF4, and shSOX2. **c** Schematic diagram of putative KLF4-binding sites in PIK3CA promoter. **d** ChIP assays on two KLF4-binding sites of PIK3CA promoter were performed in HNE-1 cells expressing shcontrol, shKLF4, and shSOX2. **e** ChIP assays on two SOX2-binding sites of PIK3CA promoter were performed in HNE-1 cells expressing shcontrol, shKLF4, and shSOX2. **f** A working model for SOX2-regulated nasopharyngeal carcinoma tumorigenesis. SOX2 recruits KLF4 binding with PIK3CA promoter to upregulate its transcription, resulting in PI3K/AKT signaling pathway activation and nasopharyngeal carcinoma tumorigenesis. Error bars ± SD. *P < 0.05. Data are representative from two independent experiments

We detected whether SOX2 regulates PIK3CA expression via interacting with KLF4. SOX2 or KLF4 depletion markedly decreased the promoter activity of PIK3CA in HNE-1 and C666-1 cell lines (Fig. 7a). Consistent with this finding, knocking down SOX2 or KLF4 in HNE-1 and C666-1 cell lines significantly decreased PIK3CA mRNA expression (Fig. 7b), suggesting that SOX2 regulated PIK3CA expression via KLF4. Moreover, we performed a silico analysis of the putative transcription factors binding to the promoter of PIK3CA (http://jaspar.genereg.net/). Two putative KLF4-binding sites were found in the promoter of PIK3CA at −338 to −329 and −224 to −215 sites (Fig. 7c). Furthermore, SOX2 depletion inhibited KLF4 binding to the PIK3CA promoter, and KLF4 knockdown similarly inhibited SOX2 binding to the PIK3CA promoter (Fig. 7d, e), indicating that SOX2 and KLF4 were dependent on each other for binding to the PIK3CA promoter. These data demonstrated that SOX2 increased PIK3CA expression by enhancing KLF4 binding to the PIK3CA gene promoter.

## Discussion

Although the mortality rate of nasopharyngeal carcinoma has been decreasing continuously due to advancement in diagnosis and treatment, nasopharyngeal carcinoma is still one of the most lethal primary malignant head and neck tumor in adults, largely because of limited knowledge of precise molecular pathogenesis and paucity of targeted therapies. Therefore, better understanding of the molecular mechanisms underlying nasopharyngeal carcinoma initiation and progression may pave the way to the development of novel preventive and therapeutic strategies. The expression of transcription factor SOX2 was widely reported upregulated in multiple types of malignant tumors, thus serving as a oncogene through regulating tumorigenesis, progression, and metastasis^9,13,14,25,26^. SOX2 was revealed to be amplified and overexpressed in gastric cancers, and depletion of SOX2 markedly inhibited cell proliferation and tumor growth in breast cancer through downregulating CCND1 and PARP^27^. Importantly, it has also been reported that SOX2 is critical for the development of the malignant phenotype of nasopharyngeal carcinoma^28,29^. However, the clear mechanisms under the SOX2 induced oncogenesis in nasopharyngeal carcinoma are still poorly understood.

In current study, SOX2 oncogenicity was further highlighted by the finding that SOX2 gene was upregulated in nasopharyngeal carcinoma based on the data derived from GEO and TCGA database. IHC staining assays also confirmed that SOX2 was overexpressed in clinical nasopharyngeal carcinoma tissues compared to that in non-cancerous tissues. Moreover, evaluation of nasopharyngeal carcinoma patient samples revealed a negative correlation between expression of SOX2 and survival of nasopharyngeal carcinoma patients. Knockdown of SOX2 by shRNAs inhibited nasopharyngeal carcinoma cell proliferation in vitro, and nasopharyngeal carcinoma tumorigenicity in vivo. Our data, taken together, demonstrated that SOX2 functions as an important oncogene in nasopharyngeal carcinoma.

Recent evidence has highlighted that SOX2 plays a vital role in the progression of multiple tumors through various mechanisms. For example, SOX2 activated lncRNA ANRIL and PVT1 by binding their promoters in nasopharyngeal carcinoma and breast cancer, respectively^10,30^. Other studies also reported that SOX2 functioned as an oncogene to regulate cancer progression by combining with FoxM1 (refs. ^31,32^). SOX2 was also shown to regulate Lin28a to activate the AKT signaling pathway thereby promoting the proliferation and maintain the self-renewal of GmGSCs-I-SB^12^. In our present study, by performing KEGG pathway analyses of the microarray gene expression data in shSOX2-HNE-1 cell line, we found that knockdown of SOX2 was significantly correlated with the PI3K/AKT signaling pathway. The western blotting assay revealed that knockdown of SOX2 markedly decreased AKT phosphorylation, which strongly suggested that SOX2 regulates nasopharyngeal carcinoma cell proliferation and tumor growth through PI3K/AKT signaling. Furthermore, we also confirmed that SOX2 binds to the promoter of PIK3CA and regulates its expression, while knockdown SOX2 significantly downregulated PIK3CA expression. Impressively, overexpression of PIK3CA effectively restored SOX2 knockdown induced inhibition of cell proliferation. Taken together, these results demonstrated that transcription factors SOX2 regulates cell proliferation in nasopharyngeal carcinoma through the PI3K/AKT signaling pathway.

Recently, SOX2 is known to interact with other transcription factors to regulate gene expression not only in embryonic stem cells (ES) but also in cancer cells^33–35^. For instance, SOX2 can interact with OCT4, a homeodomain transcription factor of the POU (PIT/OCT/UNC) family, to form Oct4/Sox2 complex, thus affecting the expression of various genes in ES cells^36^. Another study also reported that DDX17, known to be a transcription co-activator, serves as a SOX2-binding partner in ER-positive breast cancer, and then regulates the transcription activity of SOX2 (ref. ^37^). In order to explore whether similar phenomenon exists in nasopharyngeal carcinoma, a protein–protein interaction network was built according to the STRING database, which exhibited that plenty of proteins interacts with SOX2 including KLF4. Most of the studies suggested that KLF4 acts as a tumor suppressor. Upregulation of KLF4 has been reported in various tumors including esophageal^38^, gastrointestinal^18,22^, and pancreatic cancer^20,39^. Conversely, accumulating clinical and experimental evidence also demonstrated that KLF4 has a promoting effect in tumor initiation, progression, or metastasis^21,40^. Given the fact that lots of conflicting result were reported, it is important to clarify the accurate expression and underlying molecular mechanism of KLF4 in nasopharyngeal carcinoma. In our study, we confirmed that KLF4 exhibited remarkably overexpression both in clinical nasopharyngeal carcinoma specimens and sequencing data from TCGA database. We also demonstrated that knockdown of KLF4 significantly inhibited tumor growth, which further confirmed that KLF4 served as an oncogene in nasopharyngeal carcinoma. Moreover, co-IP assays showed that KLF4 bound to SOX2 directly. The ChIP-qPCR assays confirmed that knockdown of SOX2 or KLF4 inhibited each other in combination with the PIK3CA promoter, further suggesting that SOX2 and KLF4 functioned interdependently and jointly regulated the expression of PIK3CA. Taken together, these findings strongly sustained that SOX2 upregulated expression of PIK3CA through interacting with KLF4 to activate downstream PI3K/AKT signaling pathway.

In summary, as shown in genetic regulatory network depicted in Fig. 7f, we described a novel mechanism by which interaction of SOX2 and KLF4, as the transcription co-activators, significantly upregulated PIK3CA expression, thereby activating the PI3K/AKT signaling pathway, and ultimately enhanced nasopharyngeal carcinoma tumorigenesis. We expect that the results of the present study may shed light into a novel mechanism underlying the function of transcription factor SOX2 and KLF4 in nasopharyngeal carcinoma, and provide a rationale for its early detection and effective therapeutic strategies.

## Materials and methods

### Clinical tissue samples

Totally, 100 pairs of nasopharyngeal carcinoma clinical specimens and matched peritumoral specimens were collected from department of the otolaryngology in Meizhou People’ s Hospital between January 2015 and December 2016. None of those patients had received chemotherapy and/or radiotherapy before the operation. Prior patient consent and approval from the Institutional Research Ethics Committee of the Meizhou People’s Hospital were obtained before using these clinical specimens. Follow-up information of all those patients was also available. Importantly, using of these clinical specimens was carried out according to the approved guidelines of the Meizhou People’s Hospital. We have obtained the written informed consent from each patient, and all patients granted permission for the data obtained to be used in subsequent studies.

### Cell lines

C666-1, SUNE-1, HNE-1, CNE1, and CNE2 cell lines were purchased from Cell Bank of the Chinese Scientific Academy (Shanghai, China), and were cultured in Dulbecco’s modified Eagle’s medium (DMEM) (Invitrogen, Carlsbad, CA) with 10% fetal bovine serum (Gibco, Life Technologies, CA). NP69 was also purchased from Cell Bank of the Chinese Scientific Academy (Shanghai, China), and was cultured in DMEM/F12 (Invitrogen, Carlsbad, CA) containing 5% horse serum, 20 ng/ml epidermal growth factor (EGF), 10 μg/ml insulin, 100 ng/ml cholera toxin, 0.5 μg/ml hydrocortisone, and 100 μg/ml penicillin–streptomycin. Cells were cultured at 37 °C with 5% CO_2_ in a humidified incubator. By using a STR DNA fingerprinting at Shanghai Biowing Applied Biotechnology Co., Ltd (Shanghai, China), C666-1, SUNE-1, HNE-1, CNE1, CNE2, and NP69 cell lines were authenticated recently. Furthermore, we also detected the mycoplasma infection by using LookOut Mycoplasma PCR Detection kit (Sigma-Aldrich).

### Microarray assay

Total RNA was extracted from cultured cells by using the TRIzol reagent (Invitrogen, CA) and purified by using NucleoSpin® RNA clean-up Kit (Macherey-Nagel, Germany). Then, RNA integrity was evaluated under the 2100 Bioanalyser (Agilent Technologies, Palo Alto, CA, USA). Subsequently, hybridization was performed overnight on a 22K human genome array chip, V1.0 (CapitalBio Corp., Beijing, China), based on the manufacturer’s instructions. After that, the hybridization images were obtained using the LuxScan 10KA dual-channel laser scanner and digitized with LuxScan 3.0 image analysis software (CapitalBio Corp.). Finally, microarray data were collected and filtered by subtracting the background. The experiment was performed in biologic triplicates with three technical replicates. The genes dysregulated at least two-fold in comparison with the controls, with a false-discovery rate <5%, were collected for further analysis.

### IHC staining and evaluation

Antibody specific against SOX2 (Abcam) was diluted 50 times and used for the IHC of clinical nasopharyngeal carcinoma samples. The 4 μm sections were cut and incubated with primary antibody overnight. Sections were then incubated with ChemMate™ Envision™/HRP secondary antibody (Dako, Carpinteria, CA, USA) for 1 h at room temperature. After washed with phosphate-buffered saline (PBS), sections were stained with 0.5% diaminobenzidine and counterstained with Mayer’s haematoxylin.

The expression levels of SOX2 was defined semi-quantitatively by calculating the immunostaining score (IS). In brief, the proportion score was evaluated by counting the number of immune-positive cells among at least 1000 cells on each section. The staining intensity was graded as 0 (no staining), 1 (weak staining with light yellow), 2 (moderate staining with yellow–brown), or 3 (strong staining with brown). The final IS was calculated by multiplying the proportion score by the staining intensity score. According to the IS, all the clinical samples were further divided into two groups, high-SOX2 expression group and low-SOX2 expression group, based on cut-off point calculated from the mean staining index in all cases.

### ChIP-qPCR

the Chromatin Immunoprecipitation Kit (Millipore-Upstate) was used for ChIP assay according to the manufacturer’s protocol. The purified immunoprecipitated DNA and input genomic DNA were analyzed by qRT-PCR. Primers are listed in Supplementary Table 1.

### Luciferase promoter assay

For the luciferase promoter assay, pGL3-PIK3CA promoter wild type or mutant of the SOX2 binding was transfected using the Lipofectamine 2000 transfection reagent (Thermo Fisher Scientific) in accordance with the manufacturer’s protocol. pRL-TK Renilla plasmid (Promega) was used as a control. By using the Dual-Luciferase Reporter Assay kit (Promega) based on the manufacturer’s protocol, luciferase and Renilla signals in the transfected cells were detected after 48 h.

### RNA extraction and quantitative RT-PCR

Total RNA was extracted from cultured cells using Trizol reagent (Invitrogen, CA). First-strand cDNA was generated by using the PrimeScript 1^st^ Strand cDNA Synthesis Kit (TaKaRa, Dalian, China). Quantitative-PCR (qPCR) was performed in triplicate using Step One Real-Time PCR System (Applied Biosystems, Foster City, USA). *ACTB* was used as a control. Primers were listed in Supplementary Table S1.

### Cell proliferation and colony formation

Cell proliferation assay was performed using a WST-1 Assay Kit (Roche). Briefly, cells were seeded in each of the triplicate wells of a 96-well plate and incubated at 37 °C. Then, cells were split and assessed with a WST-1 assay kit. For colony formation assay in soft agar, cells were trypsinized into a single-cell suspension, and seeded in a media containing 0.4% agar on top layer and a bottom 0.8% layer per well in a 12-well plate. Then, cell culture media was changed every 3 days after seeding. Colonies were fixed and stained with 1% crystal violet solution after 2–3 weeks. The visible colony numbers scored and data were analyzed.

### Construction of vectors

The cDNA encoding Flag-KLF4, HA-SOX2, pCIG-PIK3CA, pLNCX-Myr-AKT were purchased from Addegen. KLF4 and SOX2 was PCR-amplified by Q5 High-Fidelity DNA Polymerase (BioLabs) and subcloned into the *Eco*R1 and *Xho*1 sites of the pcDNA3 vector (Invitrogen), subsequently named pCDNA3-KLF4 and pCDNA3-SOX2. pLVX-KLF4 and pLVX-SOX2 was generated from pCDNA3-KLF4 and pCDNA3-SOX2, respectively. PIK3CA promoter was amplified by PCR from normal breast tissues and sequenced, and then subcloned into pGL3 vector (Promega). Point mutations were constructed using a QuikChange Site-Directed Mutagenesis Kit (Stratagene) based on the manufacturer’s protocol. shRNAs were designed to target SOX2 (shSOX2-1 target sequence: 5′-CGAGATAAACATGGCAATCAA-3′; shSOX2-2 target sequence 5′-GTACAGTATT TATCGAGATAA-3′) and KLF4 (shKLF4-1 target sequence: 5′-GCTCCATTACCAAG AGCTCAT-3′; shKLF4-2 target sequence 5′-GCCAGAATTGGACCCGGTGTA-3′), respectively.

### Western blot analysis

Cells were lysed thoroughly in RIPA buffer (Beyotime, China), agitated for 20 min at 4 °C. Then the cell lysate was sonicated for 15 s and centrifuged at 12,000 rpm for 15 min. The protein concentration was determined using a BCA protein assay kit (Beyotime, China). Equal amounts of proteins (30 µg) were denatured and fractionated on 10% SDS-PAGE for separation. After that, the proteins were transferred to polyvinylidene difluoride membranes. After being blocked with 8% non-fat milk in TBST, the membranes were incubated with primary antibodies including KLF4 (1:1000; Cell Signaling Technology), SOX2 (1:1000; Abcam), AKT (1:1000; Cell Signaling Technology), p-AKT (1:1000; Cell Signaling Technology), and PIK3CA(1:1000; Cell Signaling Technology) at 4 °C overnight. β-Actin (1:5000, Protech) was used as the control. The membranes were then incubated with HRP-conjugated goat anti-rabbit, goat anti-mouse (Cell Signalling Technology, dilution 1:5000) secondary antibodies for 1 h at room temperature. The immunoreactive proteins were visualized with an enhanced chemiluminescence detection kit (Millipore).

### Xenografts

Briefly, BALB/c-nude mice female, 4–5 weeks old, 15–18 g (SLAC, Shanghai, China) were housed under pathogen-free conditions. HNE-1 cells were collected and washed twice with serum-free medium. After reconstituted in serum-free medium DMEM and mixed 1:1 with Matrigel (Becton-Dickinson), HNE-1 cells (2 × 10^6^) were implanted subcutaneously into the right flank of each nude mouse. All experimental procedures were carried out in accordance with institutional animal regulations and were approved by the Guidance of Institutional Animal Care and Use Committee of the Zhejiang Provincial People’s Hospital. Bioluminescence imaging was performed using the IVIS Lumina imaging station (Caliper Life Sciences).

### Statistical analysis

Statistical analyses were performed with the GraphPad Prism version 5.0 software (GraphPad). The Pearson’ s correlation coefficient was used to determine the significance of the data from patient specimens. The Student’s test or Mann–Whitney *U*-test was used to determine the significance of the in vitro and in vivo data between experimental groups. *P < 0.05 was considered statistically significant.

## Electronic supplementary material

Supplementary Figure 1

Supplementary Figure 2

Supplementary Table 1

Supplementary Table 2

Supplementary Table 3
