# Supplementary material for: SOX2 recruits KLF4 to regulate nasopharyngeal carcinoma proliferation via PI3K/AKT signaling
Source: Oncogenesis. 2018 Aug 15;7(8):61. doi: 10.1038/s41389-018-0074-2 (PMC6092437; doi:10.1038/s41389-018-0074-2)
Supplement: Supplementary file 1 — Supplementary Figure 1 [file 41389_2018_74_MOESM1_ESM.pdf]

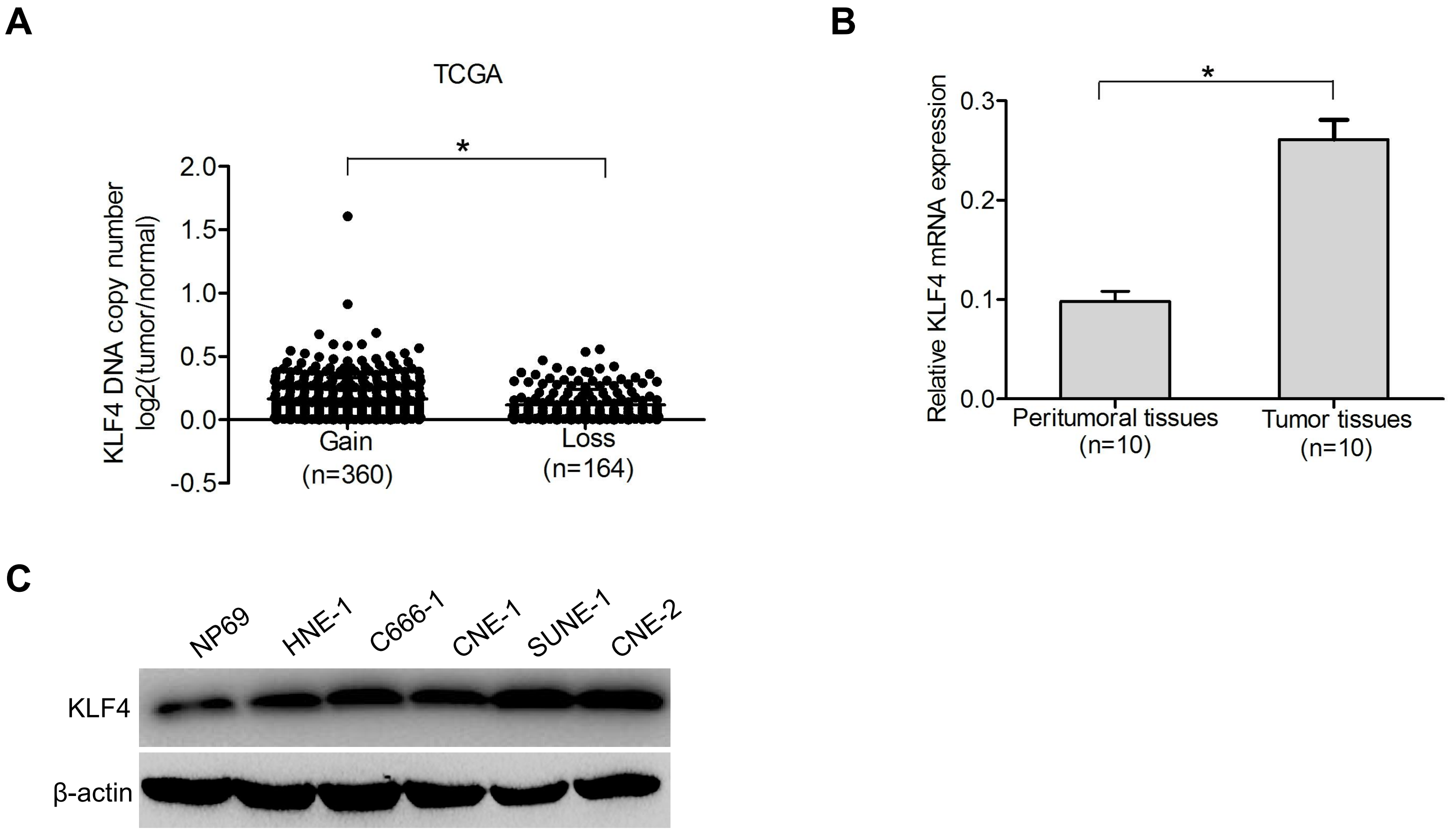

Supplemetary Figure 1 KLF4 expression is prognostic for clinical nasopharyngeal carcinoma

(A) Expression levels of KLF4 DNA copy number are significantly higher in head and neck samples compared with normal tissues from a TCGA data sets. (B) KLF4 expression in clinical nasopharyngeal tissues and peritumoral tisssues specimens. (C) Western blotting analysis of KLF4 protein expression in nasopharyngeal carcinoma cells and normal nasopharyngeal cells. Actin was used as a control.
