# Supplementary material for: SOX2 recruits KLF4 to regulate nasopharyngeal carcinoma proliferation via PI3K/AKT signaling
Source: Oncogenesis. 2018 Aug 15;7(8):61. doi: 10.1038/s41389-018-0074-2 (PMC6092437; doi:10.1038/s41389-018-0074-2)
Supplement: Supplementary file 2 — Supplementary Figure 2 [file 41389_2018_74_MOESM2_ESM.pdf]

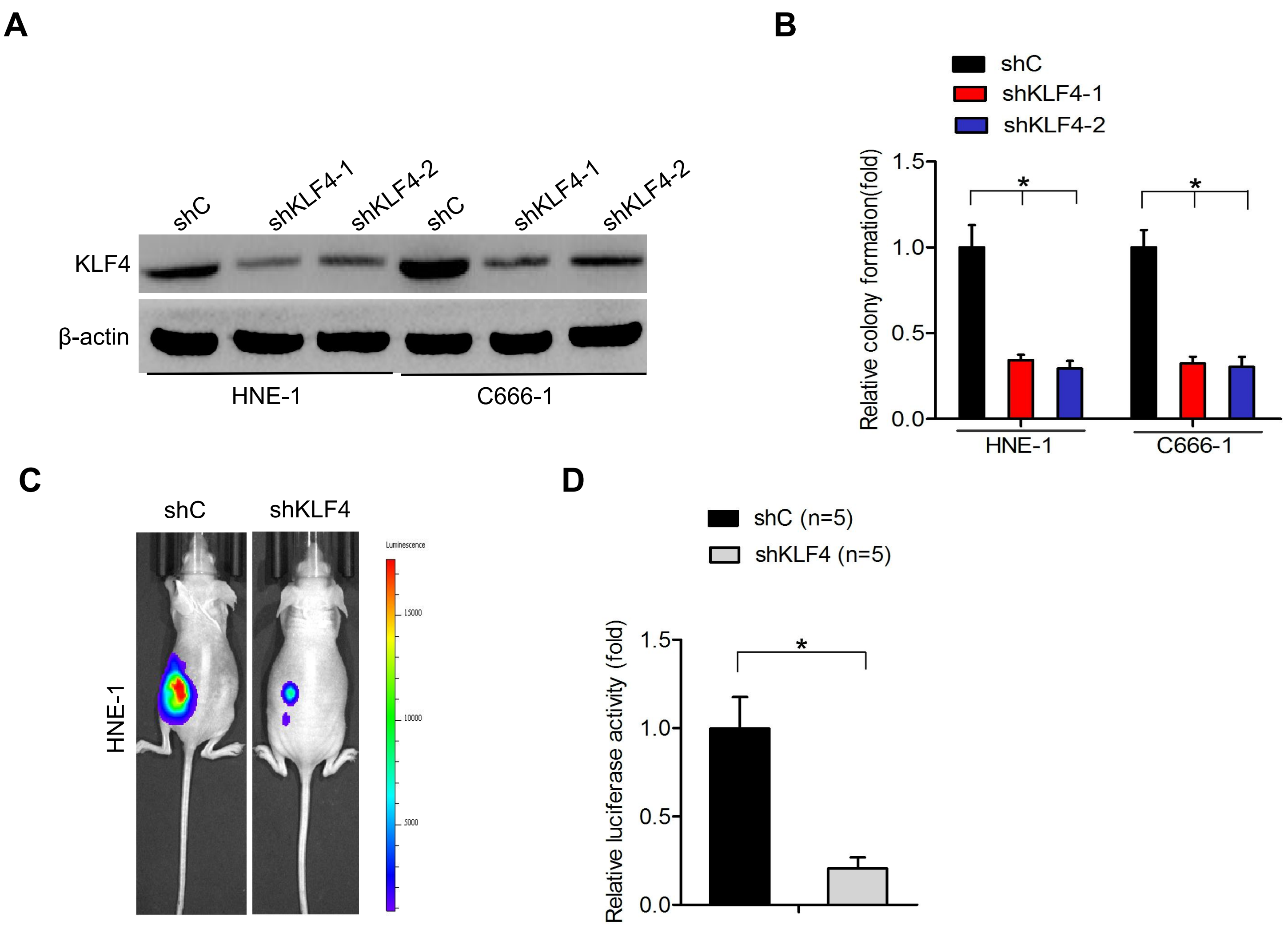

Supplemetary Figure 2 Depletion of KLF4 inhibits nasopharyngeal carcinoma growth

(A) Western blot analysis of KLF4 knockdown in HNE-1 and C666-1 cells. (B) Effects of KLF4 knockdown on nasopharyngeal carcinoma cell colony formation. (C) Representative bioluminescence images of KLF4 knockdown-inhibited HNE-1 subcutaneous tumour generation. (D) Quantification of the bioluminescence activity in C.
