# Supplementary material for: SOX2 recruits KLF4 to regulate nasopharyngeal carcinoma proliferation via PI3K/AKT signaling
Source: Oncogenesis. 2018 Aug 15;7(8):61. doi: 10.1038/s41389-018-0074-2 (PMC6092437; doi:10.1038/s41389-018-0074-2)
Supplement: Supplementary file 3 — Supplementary Table 1 [file 41389_2018_74_MOESM3_ESM.docx]

**Supplementary Table 1** Primers for qRT-PCR assays and ChIP-qPCR assays

| Primer pairs | Sequence |
| --- | --- |
| qRT-PCR,*ACTB* | 5’-CATGTACGTTGCTATCCAGGC-3’ and  5’-CTCCTTAATGTCACGCACGAT-3’ |
| qRT-PCR,*LPAR6* | 5’-TTGTATGGGTGCATGTTCAGC-3’ and  5’-GCCAATTCCGTGTTGTGAAGT-3’ |
| qRT-PCR,*SOX2* | 5’-CTCGTGCAGTTCTACTCGTCG-3’ and  5’-AGCTCTCGGTCAGGTCCTTT-3’ |
| qRT-PCR,*COL6A2* | 5’-GACTCCACCGAGATCGACCA-3’ and  5’-CTTGTAGCACTCTCCGTAGGC-3’ |
| qRT-PCR,*COL11A2* | 5’-GCTCCCCTCCTGACTCTCTAC-3’ and  5’-CCGGGTGACTCGCTTCTTG-3’ |
| qRT-PCR,*FGF13* | 5’-GTTACCAAGCTATACAGCCGAC-3’ and  5’-ACAGGGATGAGGTTAAACAGAGT-3’ |
| qRT-PCR,*ANGPT* | 5’-AACTTTCGGAAGAGCATGGAC-3’ and  5’-CGAGTCATCGTATTCGAGCGG-3’ |
| qRT-PCR,*IFNAR2* | 5’-TCATGGTGTATATCAGCCTCGT-3’ and  5’-AGTTGGTACAATGGAGTGGTTTT-3’ |
| qRT-PCR,*IGF1* | 5’-GCTCTTCAGTTCGTGTGTGGA-3’ and  5’-GCCTCCTTAGATCACAGCTCC-3’ |
| qRT-PCR,*LAMA4* | 5’-ATGAGCTGCAAGGAAAACTATCC-3’ and  5’-CTGTTTCGTTGGCTTCACTGA-3’ |
| qRT-PCR,*NOS3* | 5’-TGATGGCGAAGCGAGTGAAG-3’ and  5’-ACTCATCCATACACAGGACCC-3’ |
| qRT-PCR,*PIK3CA* | 5’-CCACGACCATCATCAGGTGAA-3’ and  5’-CCTCACGGAGGCATTCTAAAGT-3’ |
| qRT-PCR,*FGF19* | 5’-CGGAGGAAGACTGTGCTTTCG-3’ and  5’-CTCGGATCGGTACACATTGTAG-3’ |
| qRT-PCR,*YWHAG* | 5’-AGCCACTGTCGAATGAGGAAC-3’ and  5’-CTGCTCAATGCTACTGATGACC-3’ |
| qRT-PCR,*BCL2L1* | 5’-TTGCCAGCCGGAACCTATG-3’ and  5’-CGAAGGCGACCAGCAATGATA-3’ |
| qRT-PCR,KLF4 | 5’-CCCACATGAAGCGACTTCCC-3’ and  5’-CAGGTCCAGGAGATCGTTGAA-3’ |
| SOX2 shRNA #1 | 5’-TCGAGATAAACATGGCAATCAACGAATTGATTGCCATGTTTATCTCGTTTTC-3’ and  5’-TCGAGAAAACGAGATAAACATGGCAATCAATTCGTTGATTGCCATGTTTATCTCG-3’ |
| SOX2 shRNA #2 | 5’-TGTACAGTATTTATCGAGATAACGAATTATCTCGATAAATACTGTACTTTTC-3’ and  5’-TCGAGAAAAGTACAGTATTTATCGAGATAATTCGTTATCTCGATAAATACTGTAC-3’ |
| KLF4 shRNA #1 | 5’-TGCTCCATTACCAAGAGCTCATCGAAATGAGCTCTTGGTAATGGAGCTTTTC-3’and  5’-TCGAGAAAAGCTCCATTACCAAGAGCTCATTTCGATGAGCTCTTGGTAATGGAGC-3’ |
| KLF4 shRNA #2 | 5’-TGCCAGAATTGGACCCGGTGTACGAATACACCGGGTCCAATTCTGGCTTTTC-3’ and  5’-TCGAGAAAAGCCAGAATTGGACCCGGTGTATTCGTACACCGGGTCCAATTCTGGC-3’ |
| qRT-PCR,*ITGA6* | 5’-ATGCACGCGGATCGAGTTT-3’ and  5’-TTCCTGCTTCGTATTAACATGCT-3’ |
| ChIP-qPCR,PIK3CA  -2722 to -2708 | 5’-GTTTCGGTGCATATTTGAG-3’ and  5’-CGGGAGGTGAAATGTCC-3’ |
| ChIP-qPCR,PIK3CA  -2256 to -2242 | 5’-TGCTATGACACACAAC-3’ and  5’-ACAGACTTCAGCCGAC-3’ |
