# Supplementary material for: SOX2 recruits KLF4 to regulate nasopharyngeal carcinoma proliferation via PI3K/AKT signaling
Source: Oncogenesis. 2018 Aug 15;7(8):61. doi: 10.1038/s41389-018-0074-2 (PMC6092437; doi:10.1038/s41389-018-0074-2)
Supplement: Supplementary file 4 — Supplementary Table 2 [file 41389_2018_74_MOESM4_ESM.doc]

**Table S2.** Enriched pathway database from KEGG pathway analysis

| **Go term** | **Count** | ***p*** | **Protein** |  | | | | | |
| --- | --- | --- | --- | --- | --- | --- | --- | --- | --- |
| Transcriptional misregulation in cancer | 15 | 4.02E-08 | CSF2; ERG; ETV1; EWSR1; FLI1; IGF1; MEIS1; MYCN; BCL2L1; BMI1; SSX2B; UTY; HIST1H3J; RUNX2; RUNX1T1 |  | | | | | |
| Pathways in cancer | 18 | 1.59E-05 | LPAR6; TFG; RASSF1; CSF2RA; FGF13; IGF1; ITGA6; LAMA4; SMAD3; BCL2L1; SKP2; TCF7L2; TPM3;TRAF5; CUL2; IKBKG; RUNX1T1; FGF19; |  | | | | | |
| Signaling pathways regulating pluripotency of stem cells | 9 | 0.0001728 | MAPK14; IGF1; SMAD3; MEIS1; NODAL; PAX6; BMI1; BMPR1B; ACVR1B |  | | | | | |
| PI3K-Akt signaling pathway | 14 | 0.0004119 | LPAR6; COL6A2; COL11A2; FGF13; ANGPT2; IFNAR2; IGF1; ITGA6; LAMA4; NOS3; BCL2L1; YWHAG; PIK3CA; FGF19 |  | | | | | |
| HIF-1 signaling pathway | 7 | 0.0006959 | ENO3; NOX1; ANGPT2; IGF1; NOS3; NOX3; CUL2 |  | | | | | |
| TGF-beta signaling pathway | 6 | 0.0008543 | SMAD3; NODAL; PITX2; SMURF2; BMPR1B; ACVR1B |  | | | | | |
| Jak-STAT signaling pathway | 8 | 0.001548 | STAM2; CSF2; CSF2RA; IFNAR2; IL12RB2; LEP; PTPN6; BCL2L1; |  | | | | | |
| Cell cycle | 7 | 0.001735 | CDKN2D; GADD45G; SMAD3; ORC4; SKP2; YWHAG; CDC14A; |  | | | | | |
| Epstein-Barr virus infection | 9 | 0.002137 | MAPK14; POLR3B; SKP2; MAP3K7; TRAF5; YWHAG; IKBKG; ENTPD1; CD44 |  | | | | | |
| p53 signaling pathway | 5 | 0.002498 | GADD45G; IGF1; PPM1D; CCNG2;EI24 |  |  |  | | | |
| RIG-I-like receptor signaling pathway | 5 | 0.002835 | TANK; MAPK14; MAP3K7; IKBKG; ATG12 |  |  |  | | | |
| T cell receptor signaling pathway | 6 | 0.003263 | PAK4; MAPK14; CSF2; PTPN6; MAP3K7;IKBKG |  |  |  |  |  |  |
| MAPK signaling pathway | 10 | 0.003327 | GADD45G; MAP4K1; MAPK14; FGF13; IL1A; RPS6KA2; MAP3K7; CACNA1F; IKBKG; FGF19 |  |  |  |  |  |  |
| Adherens junction | 5 | 0.003401 | PVRL3; SMAD3; PTPN6; MAP3K7; TCF7L2 |  |  |  |  |  |  |
| Cell adhesion molecules | 7 | 0.004151 | CNTN1; PVRL3; HLA-DOB; ITGA6; NCAM1; NLGN3; MPZL1 |  |  |  |  |  |  |
| TNF signaling pathway | 6 | 0.004296 | MAPK14; CSF2; NOD2; MAP3K7; TRAF5; IKBKG |  |  |  |  |  |  |
| Ras signaling pathway | 9 | 0.004733 | PAK4; RASSF1; FGF13; ANGPT2; HTR7; IGF1; BCL2L1; IKBKG; FGF19 |  |  |  |  |  |  |
| ECM-receptor interaction | 5 | 0.007147 | COL6A2; COL11A2; ITGA6; LAMA4; CD44 |  |  |  |  |  |  |
| NOD-like receptor signaling pathway | 4 | 0.007896 | MAPK14; NOD2; MAP3K7; IKBKG |  |  |  |  |  |  |
| Focal adhesion | 8 | 0.008452 | PAK4; COL6A2; COL11A2; PIP5K1C; IGF1; ITGA6; LAMA4; MYL7 |  |  |  |  |  |  |
| Cytokine-cytokine receptor interaction | 9 | 0.01199 | CSF2; CSF2RA; IFNAR2; IL1A; IL12RB2; LEP; BMPR1B; IL18RAP; ACVR1B |  |  |  |  |  |  |
| Hippo signaling pathway | 6 | 0.02032 | RASSF1; SMAD3; NF2; BMPR1B; TCF7L2; YWHAG |  |  |  |  |  |  |
| NF-kappa B signaling pathway | 4 | 0.03713 | BCL2L1; MAP3K7; TRAF5; IKBKG |  |  |  |  |  |  |
| Ubiquitin mediated proteolysis | 5 | 0.04149 | PPIL2; SMURF2; SKP2; UBE2D3; CUL2 |  |  |  |  |  |  |
