# Supplementary material for: SOX2 recruits KLF4 to regulate nasopharyngeal carcinoma proliferation via PI3K/AKT signaling
Source: Oncogenesis. 2018 Aug 15;7(8):61. doi: 10.1038/s41389-018-0074-2 (PMC6092437; doi:10.1038/s41389-018-0074-2)
Supplement: Supplementary file 5 — Supplementary Table 3 [file 41389_2018_74_MOESM5_ESM.doc]

**Table S4.** Main genes differentially expressed in nasopharyngeal carcinoma cell line-HNE-1 upon SOX2 knock-down

| **Gene symbol** | **GenBank** | **Gene name** | **Microarray data** | **KGEE pathway database** |
| --- | --- | --- | --- | --- |
| *LPAR6* | NM_001162498 | lysophosphatidic acid receptor 6 | D | PI3K-Akt signaling pathway |
| *COL6A2* | NM_058174 | collagen type VI alpha 2 chain | D | PI3K-Akt signaling pathway |
| *COL11A2* | NM_001163771 | collagen type XI alpha 2 chain | D | PI3K-Akt signaling pathway |
| *FGF13* | NM_033642 | fibroblast growth factor 13 | D | PI3K-Akt signaling pathway |
| *ANGPT2* | NM_001147 | angiopoietin 2 | D | PI3K-Akt signaling pathway |
| *IFNAR2* | NM_207585 | interferon alpha and beta receptor subunit 2 | D | PI3K-Akt signaling pathway |
| *IGF1* | NM_000618 | insulin like growth factor 1 | D | PI3K-Akt signaling pathway |
| *ITGA6* | NM_001079818 | integrin subunit alpha 6 | U | PI3K-Akt signaling pathway |
| *LAMA4* | NM_001105207 | laminin, alpha 4 | D | PI3K-Akt signaling pathway |
| *NOS3* | NM_001160109 | nitric oxide synthase 3 | D | PI3K-Akt signaling pathway |
| *BCL2L1* | NM_001191 | BCL2 like 1 | U | PI3K-Akt signaling pathway |
| *YWHAG* | NM_012479 | tyrosine 3-monooxygenase/tryptophan 5-monooxygenase activation protein gamma | U | PI3K-Akt signaling pathway |
| *PIK3CA* | NM_006218 | phosphatidylinositol-4,5-bisphosphate 3-kinase catalytic subunit alpha | D | PI3K-Akt signaling pathway |
| *FGF19* | NM_005117 | fibroblast growth factor 19 | D | PI3K-Akt signaling pathway |

D, down-regulated genes; U, up-regulated genes.
